# The impact of educational concerns and satisfaction on baccalaureate nursing students’ distress and quality of life during the Covid-19 pandemic; a cross-sectional study

**DOI:** 10.1186/s12912-022-00962-7

**Published:** 2022-07-11

**Authors:** Tone Nygaard Flølo, Kari Hanne Gjeilo, John Roger Andersen, Kristin Haraldstad, Inger Helene Hardeland Hjelmeland, Marjolein Memelink Iversen, Borghild Løyland, Tone Merete Norekvål, Kirsti Riiser, Gudrun Rohde, Kristin Hjortland Urstad, Inger Utne, Elisabeth Grov Beisland, on behalf of the LIVSFORSK network, Tone Nygaard Flølo, Kari Hanne Gjeilo, John Roger Andersen, Kristin Haraldstad, Inger Helene Hardeland Hjelmeland, Marjolein Memelink Iversen, Borghild Løyland, Tone Merete Norekvål, Kirsti Riiser, Gudrun Rohde, Kristin Hjortland Urstad, Inger Utne, Elisabeth Grov Beisland

**Affiliations:** 1 Department of Nursing and Health Promotion, Faculty of Health Sciences, Oslo Metropolitan University, P.O. Box 4, St. Olavs plass, NO-0130 Oslo, Norway; 2 Department of Surgery, Voss Hospital, Haukeland University Hospital/The Western Norway Health Region Authority, Sjukehusvegen 16, 5704 Voss, Norway; 3 Department of Public Health and Nursing, NTNU, Norwegian University of Science and Technology, NO-7491 Trondheim, Norway; 4 Department of Cardiology and Department of Cardiothoracic Surgery, St. Olav Hospital, Trondheim University Hospital, NO-7006, Postbox 3250, Torgarden, Trondheim, Norway; 5 Department of Health and Caring Sciences, Western Norway University of Applied Sciences, Inndalsveien 28, 5063 Kronstad, Bergen, Norway; 6 Førde Hospital Trust, Post Office Box 1000, N-6807 Førde, Norway; 7 Department of Health and Nursing Sciences, University of Agder, P.O Box 422, 4604 Kristiansand, Norway; 8 Department of Heart Disease, Haukeland University Hospital, Jonas Lies vei 65, NO-5021 Bergen, Norway; 9 Department of Physiotherapy, Faculty of Health Sciences, Oslo Metropolitan University, P.O. Box 4, St. Olavs plass, NO-0130 Oslo, Norway; 10 Department of Clinical Research, Sorlandet Hospital, SSHF, P.O. Box 416, 4604 Kristiansand, Norway; 11 Department of Quality and Health Technology, University of Stavanger, Kjell Arholms gate 41, 4021 Stavanger, Norway

**Keywords:** Student satisfaction, Academic quality concerns, Quality of life, Psychological distress, Nursing students

## Abstract

**Background:**

High levels of psychological distress and poor overall quality of life (QOL) have been identified among nursing students during the COVID-19 pandemic. The pandemic necessitated improvised reconstructions of educational curriculums and restrictions in clinical placement and training at campuses, possibly reducing educational quality.

**Objectives:**

We explored whether baccalaureate nursing students’ concerns and satisfaction with the educational curriculum, focusing on the conduct of clinical training, were associated with perceived psychological distress and overall QOL.

**Methods:**

Baccalaureate nursing students (*N*=6088) from five Norwegian universities were invited to an internet-based, cross-sectional survey during the second wave of the pandemic. The survey included COVID-19 specific questions on health, education and clinical training, the Fear of COVID-19 scale (FCV-19S), The Hopkins Symptom Checklist (SCL-5) and overall QOL. Data from national surveys on satisfaction with the educational curriculum, before and during the pandemic were used for comparison.

**Results:**

In total, 2605 (43%) students responded, of whom 1591 (61%) had been engaged in clinical training during the pandemic. Overall, 53% were either satisfied or fully satisfied with their educational curriculum, with the level of satisfaction being significantly lower than pre-pandemic reference values. Also, 79% were concerned or highly concerned about the educational quality. In multiple regression analyses for all students, lower levels of satisfaction and higher levels of quality concerns were associated with worse SCL-5 scores. Furthermore, satisfaction with the educational curriculum was positively associated with overall QOL. For students engaged in clinical training, only *concerns about infecting others* were additionally associated with psychological distress. None of the items related to clinical training were associated with overall QOL.

**Conclusion:**

Nursing students’ educational satisfaction and quality concerns may significantly impact perceived psychological distress and overall QOL during a pandemic. However, with necessary adaptations implemented, concerns regarding the conduct of clinical training account for little of these associations.

## Introduction

During the coronavirus disease 2019 (COVID-19) pandemic, elevated levels of psychological distress and reduced overall quality of life (QOL) have been identified among students in higher education, globally and in Norway [1–3]. A Norwegian survey during the pandemic found a general decline in measures of life-satisfaction and mental health with young adults and students more severely affected [4]. Yet, during the second wave of the pandemic (January and February 2021) we observed that fear of COVID-19 in Norwegian nursing students accounted for only minor parts of the deteriorated psychological health and overall QOL [3], suggesting that other factors related to being a student during a pandemic may contribute to the observed changes. Identifying education-related predictors of students’ well-being throughout the course of the pandemic may be important for adequate handling of restrictions in higher education institutions.

College and university education, *i.e*. the years where mostly young adults aim to acquire knowledge or skills that will be decisive for their professional lives, is recognized as a high-stress period [5], and psychological distress may lead to poor academic performance [6, 7]. For instance, in undergraduate students prior to the COVID-19 crisis, academic performance, pressure to succeed, and post-graduation plans were the students’ top three concerns [8]. For nursing students in particular, psychological distress has been identified to negatively impact QOL, educational and clinical training performance [9].

For educational institutions, the pandemic necessitated improvised reconstructions of curriculums, rapid move to online distance learning, and restrictions in practical placement or simulation training both in and outside of campuses, all possibly inducing additional stress [10]. Such changes were implemented with similar rigidity during the second wave of the pandemic beginning in Norway in November 2020. Thus, finding ways to mitigate psychological distress and sustain overall QOL may benefit students’ learning and society’s need for competent future professionals.

Student satisfaction has gained global attention as a measure of effectiveness of educational curriculums [11]. Instructional effectiveness, student support facilities, internet and library access, administrative staff efficiency, university environment, and student characteristics such as gender, ethnicity, and age are all factors identified to impact overall student satisfaction in higher education [12–15]. Furthermore, empirical analysis and deconstruction of the concept *student satisfaction* suggest academic and pedagogic quality of teaching to be crucial determinants of student satisfaction, potentially overlapping with students’ assessment of teaching [16]. In Norway, aspects of students’ views on educational quality, including overall student satisfaction are assessed by national surveys annually [17].

Nursing students may face higher levels of stress than other health professional students, even under normal circumstances [18, 19] and may therefore be particularly vulnerable to educational constraints resulting from measures to restrict spread of COVID-19. The conduct of clinical training, essential in all nursing educations and accounting for up to 50% of most European and international curriculums [20, 21] may be particularly difficult during a pandemic. For a three-year baccalaureate education, restrictions beginning in March 2020 will for some students have been operative for up to half of the educational period. Hence, nursing students expressed concerns about interruptions in their clinical training and how these would affect overall quality of education and post-graduation careers [22].

The purpose of this study was two-fold. First, we determined baccalaureate nursing students’ satisfaction with their educational curriculum during the second wave of the COVID-19 pandemic, compared to pre-pandemic reference data. Second, we investigated how students’ satisfaction was related to concerns about the education quality, and finally how these two measures were associated with psychological distress and overall QOL.

## Methods

### Setting and design

Full- and part-time baccalaureate nursing students (N=6088) from five Norwegian universities at ten campuses were invited to participate in an internet-based cross-sectional survey from January 27^th^ to February 28^th^ 2021, at the peak of the second wave of the pandemic in Norway. Participating universities were the Norwegian University of Science and Technology, University of Agder, University of Stavanger, Western Norway University of Applied Sciences and Oslo Metropolitan University. Detailed information about study setting, design and data collection were recently published [3]. Two national surveys on satisfaction with the educational curriculum were used for comparison: The National Students Survey 2019 (pre-pandemic results) and The National Students Survey 2020 (October 2020 prior to the second pandemic wave in Norway) [17].

### Measures

Respondents provided information on study site, year of study, age, and household status. The complete survey included COVID-19 specific questions related to health, education and clinical training, the Fear of COVID-19 scale (FCV-19S), the Hopkins Symptom Checklist (SCL-5) and overall quality of life (QOL) [23–25]. Additional COVID-19 specific questions about students’ concerns related to clinical training during societal lockdown were collected only from students who engaged in such training.

Feasibility pilot studies of the complete questionnaire as well as content and validity of all included questionnaires and COVID-19-specific items are detailed previously [3]. Psychological distress was measured with the SCL-5, encompassing five items rated on a scale from 1 “not at all” to 5 “extremely” [23, 26]. Average item score was determined by dividing total score by number of items answered [27]. Higher scores indicate greater psychological distress. Overall QOL was captured by one question, *All in all, how satisfied are you with your life at this time?*, representing an adapted version of the Cantril Ladder [24]. Answers were scored from 0 (not at all satisfied) to 10 (highly satisfied), with scores of 6 or more indicating high life satisfaction [28].

Specific to the present analysis, global satisfaction with the educational curriculum was measured with one item retrieved from the Norwegian National Student Survey, *I am, overall, satisfied with the curriculum I am currently attending* [17]. Concerns about the quality of the education were measured with one item, *Due to the COVID-19 pandemic I am concerned that the quality of my education will be poorer than it would otherwise have been*. Additional questions were related to the conduct of clinical training and placements and included the students’ perceived risk of being infected with COVID-19, necessary knowledge of infection control, concerns about infecting others, concerns about absenteeism, concerns about completion, experience of fewer learning situations and experience with insufficient guidance. Responses to all these questions were rated from 1 (strongly disagree) to 5 (strongly agree). Further, the students were asked if they had been in self-imposed quarantine during the pandemic.

### Ethics

Participating students consented by completing and submitting the electronic survey in “SurveyXact” (https://www.surveyxact.com/). Their answers were stored anonymously, hence ethical approval was not required according to Norwegian legislation. However, the Data Protection Officer at Western Norway University of Applied Sciences evaluated the survey, and additional approval was obtained from each University.

### Statistics

Categorical variables are expressed as numbers and percentages, and continuous variables as means and standard deviations (SD). Mean scores for satisfaction with the curriculum were available from 2019 (prior to the COVID-19 pandemic) and from October/November 2020 (prior to/at the beginning of the second wave of the pandemic) for nursing students at the five participating universities [17]. For comparison, the mean scores were adjusted according to the relative contribution of each university in the present study, and compared using one sample t-test [29]. For binary logistic regression analysis, satisfaction with the curriculum and concerns about the quality of the education were dichotomized and used as dependent variables. Variables with univariate significant associations were entered in multivariate analyses and results expressed as odds ratios (OR) and 95% confidence intervals (CI). Level of explained variance in the models were expressed as Nagelkerke pseudo-R^2^ values. Hierarchical regression analyses, with study site as clusters, were conducted to investigate the impact of satisfaction and quality concerns on SCL-5 and QOL used as z-transformed continuous variables. Variables previously identified to be independently associated with either SCL-5 or QOL were entered in the models together with satisfaction and concerns related to the education, both used in their original 5-point ordinal scale.

Both binary logistic and linear hierarchical regression analyses were performed first on all students. The analyses were repeated for students who engaged in clinical training during the societal lock-down with COVID-19 specific questions included as independent variables.

In the hierarchical regression analyses, effect-size of the associations were interpreted from the change in the dependent variable per 2 SD changes in FCV-19S or between respondents representing the lower or higher end of the discrete variables with 2–5 categories [30, 31]. In t-tests, the effect-size was estimated as Cohen’s d [32]. Overall, effect-sizes were interpreted as follows: trivial (< 0.2), small (0.2 to < 0.5), moderate (0.5 to < 0.8) and large (≥ 0.8). All tests were two-sided and *p*-values below 0.05 were considered significant.

## Results

In total, 2605 of the 6088 students responded to the survey, yielding a response rate of 43%, differing between the universities from 21 to 50%. Among these, 1591 (61%) reported to have been engaged in clinical training, either in primary or specialist care, including community-based and institutional services, during the pandemic. Cronbach’s alpha for FCV-19S was 0.87 (ranging from 0.84 to 0.86 if singe items were deleted), identical for the total sample and those engaged in clinical training, only. For SCL-5, Cronbach’s alpha was 0.88 (ranging from 0.84 to 0.87 if single items were deleted) both for the total sample and those engaged in clinical training.

### Respondents’ satisfaction with their educational curriculum

Overall, 54% of the students were either satisfied (n=1108, 43%) or fully satisfied (n=274, 11%) with their curriculum (Table 1). The proportion of either satisfied of fully satisfied students varied from 42 to 72% among the five participating universities. The mean score for the whole group of respondents was 3.40 (SD 1.01). Compared to the adjusted mean score for satisfaction obtained for bachelor nursing students at the same universities in 2019 (3.74) and in October/November 2020 (3.82) the mean score of the respondents was 0.34 (*p*<0.001) and 0.42 (*p*<0.001) lower, both corresponding to small effect-sizes.

**Table 1:** Student characteristics associated with satisfaction with the educational curriculum. Multivariate binary logistic regression analysis with satisfaction with the educational curriculum^a^ as the dependent variable

		All students (***n***=2605)				Students engaged in clinical training (***n***=1591)		
Variables	n (%)	Odds ratio	95% Confidence Interval	*P*-value	n (%)	Odds ratio	95% Confidence Interval	*P*-value^c^
**University**								
NTNU^b^	212 (8)	Ref.		**< 0.001**	126 (8)	Ref.		**< 0.001**
Agder	396 (15)	2.434	1.399, 4.341		246 (15)	5.541	2.514, 12.209	
Stavanger	183 (7)	0.734	0.419, 1.288		115 (7)	0.942	0.452, 1.962	
Western Norway	873 (34)	1.740	1.098, 2.756		592 (34)	2.841	1.550, 5,209	
OsloMet	937 (36)	1.156	0.742, 1.801		512 (36)	1.412	0.790 2.524	
**Number of times tested for COVID-19**								
Never	765 (29)			0.410	403 (25)			0.236
1	724 (28)	0.803	0.553, 1.166		445 (28)	0.902	0.580, 1.403	
2	445 (17)	1.160	0.773, 1.741		292 (18)	1.513	0.911, 2.513	
3	326 (12)	1.117	0.772, 1.615		208 (13)	1.399	0.807, 2.424	
≥4	346 (13)	0.905	0.659, 1.241		243 (15)	0.660	0.677, 1.851	
**Trust in governmental handling of the COVID-19 situation**								
Strongly disagree	154 (6)	Ref.		0.063	90 (6)	Ref.		0.083
Disagree	77 (3)	0.602	0.300, 1.208		40 (3)	0.648	0.253, 1.847	
Neither disagree nor agree	562 (22)	0.932	0.580, 1.499		312 (20)	0.802	0.417, 1.545	
Agree	1344 (52)	1.257	0.798, 1.981		847 (53)	1.333	0.714, 2.488	
Strongly agree	469 (18)	1.169	0.683, 2.000		302 (19)	1.280	0.606, 2.704	
**Trust in universities’ handling of the COVID-19 situation**								
Strongly disagree	181 (7)	Ref.		**< 0.001**	122 (8)	Ref.		**< 0.001**
Disagree	447 (17)	4.981	3.288, 7.546		276 (17)	4.773	2.766, 8.234	
Neither disagree nor agree	783 (30)	15.215	9.994, 23.163		475 (30)	14.031	8.035, 24.502	
Agree	982 (38)	44.106	27.394, 71.012		584 (37)	48.904	25.468, 93.908	
Strongly agree	213 (8)	61.319	27.014, 139.189		134 (8)	164.892	35.171, 773.086	
**Feeling lonely due to COVID-19**								
Strongly disagree	164 (6)	Ref.		0.672	109 (7)	Ref.		0.670
Disagree	380 (15)	1.273	0.686, 2.359		251 (16)	1.075	0.472, 2.446	
Neither disagree nor agree	444 (17)	1.388	0.765, 2.517		292 (18)	1.203	0.542, 2.667	
Agree	899 (34)	1.100	0.638, 1.899		550 (35)	0.973	0.466, 2.029	
Strongly agree	718 (28)	1.079	0.616, 1.891		389 (24)	1.310	0.613, 2.801	
**Concerns about the quality of education**								
Strongly agree	1196 (46)	Ref		**< 0.001**		Ref		**0.001**
Agree	869 (33)	2.069	1.015, 4.219			1.942	1.311, 2.878	
Neither disagree nor agree	287 (11)	2.253	1.144, 4.435			3.097	1.405, 6.827	
Disagree	160 (6)	4.519	2.380, 8.580			1.467	0.658, 3.267	
Strongly disagree	94 (4)	2.164	1.626, 2.879			3.622	1.266, 10.364	
**Fear of COVID-19 (continuous z-score)**	2605 (100)	1.056	0.936, 1.190	0.378				
**Clinical training during the pandemic**				0.689				
Yes	1591 (61)	Ref.						
No	1014 (39)	1.042	0.852, 1.275					
**Concerns about high absenteeism during clinical training**								
Strongly agree					810 (51)	Ref.		**0.013**
Agree					418 (26)	1.112	0.691, 1.791	
Neither agree nor disagree					138 (9)	1.277	0.642, 2.541	
Disagree					119 (7)	3.051	1.205, 7.723	
Strongly disagree					68 (4)	0.371	0.151, 0.910	
**Concerns about the completion of clinical training**								
Strongly agree					935 (59)	Ref.		0.465
Agree					445 (28)	0.909	0.559, 1.479	
Neither agree nor disagree					93 (6)	0.610	0.278, 1.988	
Disagree					50 (3)	0.631	0.201, 1.988	
Strongly disagree					30 (2)	2.932	0.441, 19.497	
**Fewer learning situations during clinical training**								
Strongly agree					476 (30)	Ref.		**0.007**
Agree					452 (28)	1.803	1.170, 2.778	
Neither agree nor disagree					268 (17)	2.279	1.359, 3.823	
Disagree					215 (14)	1.058	0.588, 1.903	
Strongly disagree					141 (9)	1.420	0.641, 3.146	
**Insufficient guidance during clinical training**								
Strongly agree					195 (12)	Ref.		0.609
Agree					264 (17)	1.174	0.684, 2.012	
Neither agree nor disagree					396 (25)	1.427	0.838, 2.428	
Disagree					434 (27)	1.465	0.831, 2.583	
Strongly disagree					273 (17)	1.547	0.778, 3.075	

^a^Recoded and dichotomized to high and low satisfaction; ^b^Norwegian University of Science and Technology; ^c^P-values below 0.05 in bold

For logistic regression analyses satisfaction was dichotomized into low (response levels 1-3) and high (levels 4-5, Table 1). In multivariate analysis including all students, study site, the level of trust in universities’ handling of the COVID-19 situation (strongly agree versus strongly disagree, OR 61.3) and concerns about the quality of the education during the pandemic (disagree versus strongly agree, OR 4.5) were significantly associated with the level of satisfaction. There was no difference between students engaged in clinical training during the pandemic and those not involved (OR 1.042, *p*=0.689). The Nagelkerke pseudo-R^2^ value for the final model was 0.41.

To explore the added significance of students’ experience during clinical training, the eight thereto related questions were added in analyses for the 1591 respondents engaged in clinical training during the pandemic. Again, there were significant associations with study site, trust in the institutions’ handling (strongly agree versus strongly disagree, OR 164.9) and quality concerns (strongly disagree versus strongly agree, OR 3.6). Items related to clinical training, and significantly associated with satisfaction, were concerns about absenteeism (disagree versus strongly agree, OR 3.0) and the experience of fewer learning situations (neither agree nor disagree versus strongly agree, OR 2.3). The Nagelkerke pseudo-R^2^ value for the final model in the subset of students with clinical training, increased to 0.44.

Sensitivity analyses using an alternative dichotomization (1-2 versus 3-5) gave essentially identical results.

### Respondents’ concerns about the quality of education during the pandemic

Overall, 79% of the students reported to be concerned (*n*=869, 33%) or highly concerned (n=1196, 46%) about the educational quality during the pandemic (Table 2). The proportion of concerned or highly concerned nursing students ranged from 70% to 82% between universities.

**Table 2:** Student characteristics associated with concerns about the quality of the nursing education. Multivariate binary logistic regression analysis with concerns about quality of the baccalaureate nursing education^**a**^ as the dependent variable

		All students (***n***=2605)				Students engaged in clinical training (***n***=1591)		
Variables	n (%)	Odds ratio	95% Confidence Interval	*P*-value	n (%)	Odds ratio	95% Confidence Interval	*P*-value^c^
**University**								
NTNU^b^	212 (8)	Ref.		**0.008**	126 (8)	Ref.		**0.047**
Agder	396 (15)	0.775	0.501, 1.199		246 (15)	0.631	0.345, 1.156	
Stavanger	183 (7)	1.067	0.618, 1.844		115 (7)	1.060	0.493, 2.277	
Western Norway	873 (34)	1.337	0.884, 2.022		592 (34)	1.211	0.688, 2.129	
OsloMet	937 (36)	0.930	0.617, 1.401		512 (36)	0.918	0.518, 1.626	
**Year of study**								
1st	1073 (41)	Ref.		**< 0.001**	173 (11)	Ref.		**<0.001**
2nd	800 (31)	0.673	0.461, 0.982		738 (46)	0.636	0.346, 1.171	
3rd	728 (28)	0.416	0.285, 0.606		680 (43)	0.316	0.171, 0.582	
**Age category**								
<25	1845 (71)	Ref.		**< 0.001**	1075 (68)	Ref.		0.083
25-29	374 (14)	0.708	0.530, 0.945		262 (16)	0.675	0.463, 0.985	
30+	382 (15)	0.585	0.445, 0.769		252 (16)	0.754	0.511, 1.112	
**Quarantine status related to COVID-19**								
Never	1302 (50)	Ref.		0.172	768 (48)	Ref.		0.223
Present	49 (2)	1.164	0.939, 1.442		29 (2)	3.216	0.829, 12.475	
Previous	1255 (48)	1.901	0.779, 4.644		794 (50)	0.994	0.739, 1.335	
**Trust in governmental handling of the COVID-19 situation**								
Strongly disagree	154 (6)	Ref.		0.134	90 (6)	Ref.		0.707
Disagree	77 (3)	0.406	0.180, 0.920		40 (3)	0.604	0.181, 2.011	
Neither disagree nor agree	562 (22)	0.552	0.305, 0.998		312 (20)	0.575	0.251, 1.381	
Agree	1344 (52)	0.693	0.395, 1.216		847 (53)	0.713	0.326, 1.559	
Strongly agree	469 (18)	0.679	0.377, 1.225		302 (19)	0.690	0.305, 1.559	
**Trust in universities’ handling of the COVID-19 situation**								
Strongly disagree	181 (7)	Ref.		**< 0.001**	122 (8)	Ref.		**0.001**
Disagree	447 (17)	0.938	0.474, 1.854		276 (17)	0.890	0.368, 2.152	
Neither disagree nor agree	783 (30)	0.839	0.433, 1.627		475 (30)	0.838	0.350, 2.004	
Agree	982 (38)	0.463	0.240, 0.892		584 (37)	0.521	0.217, 1.253	
Strongly agree	213 (8)	0.299	0.146, 0.613		134 (8)	0.279	0.105, 0.744	
**Feeling lonely due to COVID-19**								
Strongly disagree	164 (6)	Ref.		**< 0.001**	109 (7)	Ref.		**<0.001**
Disagree	380 (15)	1.561	1.031, 2.362		251 (16)	1.486	0.848, 2.602	
Neither disagree nor agree	444 (17)	1.880	1.242, 2.847		292 (18)	1.799	1.027, 3.152	
Agree	899 (34)	3.252	2.183, 4.843		550 (35)	2.801	1.638, 4.791	
Strongly agree	718 (28)	5.219	3.315, 8.215		389 (24)	5.555	2.934, 10.516	
**Satisfaction with the curriculum**								
Strongly agree	274 (11)	Ref.		**< 0.001**	182 (11)	Ref.		**0.001**
Agree	1108 (43)	1.619	1.169, 2.241		696 (44)	1.351	0.863, 2.115	
Neither agree nor disagree	731 (28)	2.353	1.596, 3.468		408 (26)	1.946	1.122, 3.372	
Disagree	365 (14)	5.161	2.927, 9.103		225 (14)	3.909	1.815, 8.419	
Strongly disagree	128 (5)	2.081	1.038, 4.171		80 (5)	0.934	0.360, 2.419	
**Fear of COVID-19 (continuous z-score)**	2605 (100)	1.044	0.929, 1.174	0.470	1590 (100)	0.874	0.730, 1.046	0.141
**Clinical training during the pandemic**								
Yes	1591 (61)	Ref.		0.150				
No	1014 (39)	0.773	0.545, 1.097					
**Concerns about getting infected during clinical training**								
Strongly agree					398 (25)	Ref.		0.626
Agree					555 (35)	1.016	0.654, 1.578	
Neither agree nor disagree					237 (15)	0.727	0.428, 1.234	
Disagree					249 (16)	0.893	0.516, 1.546	
Strongly disagree					114 (7)	0.785	0.396, 1.553	
**Necessary knowledge of infection control**								
Strongly agree					367 (23)	Ref.		0.199
Agree					859 (54)	1.254	0.869, 1.808	
Neither agree nor disagree					222 (14)	0.993	0.602, 1.638	
Disagree					86 (5)	1.457	0.689, 3.083	
Strongly disagree					19 (1)	0.412	0.137, 1.243	
**Concerns about infecting others during clinical training**								
Strongly agree					788 (50)	Ref.		0.525
Agree					548 (34)	0.886	0.621, 1.265	
Neither agree nor disagree					94 (6)	0.677	0.363, 1.265	
Disagree					78 (5)	0.684	0.360, 1.299	
Strongly disagree					45 (3)	0.557	0.224, 1.384	
**Self-imposed quarantine during clinical training**								
No					841 (53)			0.390
Yes					712 (47)	1.142	0.843, 1.546	
**Concerns about high absenteeism during clinical training**								
Strongly agree					810 (51)	Ref.		0.186
Agree					418 (26)	1.123	0.734, 1.716	
Neither agree nor disagree					138 (9)	1.718	0.927, 3.187	
Disagree					119 (7)	1.485	0.796, 2.769	
Strongly disagree					68 (4)	2.339	1.008, 5.424	
**Concerns about completion of clinical training**								
Strongly agree					935 (59)	Ref.		**0.002**
Agree					445 (28)	0.667	0.440, 1.011	
Neither agree nor disagree					93 (6)	0.518	0.278, 0.966	
Disagree					50 (3)	0.242	0.111, 0.527	
Strongly disagree					30 (2)	0.271	0.095, 0.771	
**Fewer learning situations during clinical training**								
Strongly agree					476 (30)	Ref.		**< 0.001**
Agree					452 (28)	0.554	0.344, 0.892	
Neither agree nor disagree					268 (17)	0.326	0.193, 0.551	
Disagree					215 (14)	0.219	0.129, 0.373	
Strongly disagree					141 (9)	0.128	0.068, 0.242	
**Insufficient guidance during clinical training**								
Strongly agree					195 (12)	Ref.		0.186
Agree					264 (17)	1.296	0.625, 2.684	
Neither agree nor disagree					396 (25)	0.946	0.480, 1.864	
Disagree					434 (27)	0.701	0.362, 1.358	
Strongly disagree					273 (17)	0.915	0.448, 1.870	

^a^Recoded and dichotomized into high and low levels of quality concerns; ^b^Norwegian University of Science and Technology; ^c^*P*-values below 0.05 in bold

For logistic regression analyses of factors associated with level of concern, the responses were dichotomized into low (1-3) and high (4-5, Table 2). For all students, study site, year of study (third year versus first, OR 0.42), age (oldest versus youngest category, OR 0.59), the level of trust in universities’ handling of the COVID-19 situation (strongly agree versus strongly disagree, OR 0.3), feeling of loneliness (strongly agree versus strongly disagree, OR 5.2) and satisfaction with the curriculum (disagree versus strongly agree, OR 5.2) were significant in multivariate analysis, for a Nagelkerke pseudo-R^2^ value of 0.25. There was no difference between students engaged in clinical practice during the pandemic and those not involved (OR 0.773, *p*=0.150).

Adding the questions related to clinical training during the pandemic, the logistic regression analyses were repeated for the 1591 respondents engaged in clinical training. Again, in multivariate analysis, there were significant associations with study site, year of study (third year versus first, OR 0.32), trust in the institutions’ handling (strongly agree versus strongly disagree, OR 0.28), feeling of loneliness (strongly agree versus strongly disagree, OR 5.6) and satisfaction (disagree versus strongly agree, OR 3.9). All items related to clinical training were univariately associated with quality concerns, but only concerns for completion of clinical training (disagree versus strongly agree, OR 0.24) and the experience of fewer learning situations (strongly disagree versus strongly agree, OR 0.13) retained significance in multivariate analysis. The Nagelkerke pseudo-R^2^ value for the final model in the subset of students with clinical training increased to 0.37.

Sensitivity analyses using alternative dichotomization (1-4 versus 5) gave similar results.

### Impact of satisfaction and quality concerns on psychological distress

The association of student reported satisfaction with the educational curriculum and concerns about the quality of the nursing education on SCL-5 scores, were explored in all students and those engaged in clinical training during the pandemic. The hierarchical regression models using SCL-5 scores as the dependent variable for all students is presented in Table 3. For all students, both satisfaction (effect-size for strongly disagree versus strongly agree = 0.39, *p*<0.001) and quality concerns (effect-size for strongly disagree versus strongly agree = 0.17, *p*=0.031) were significantly associated with SCL-5 scores, *i.e.* lower levels of satisfaction and higher levels of concerns were associated with worse SCL-5 scores. For students involved in clinical training during the pandemic, including additionally items related to clinical training in the model, only satisfaction retained significance (effect-size for strongly disagree versus strongly agree = 0.36, *p* = 0.007), whereas the level of concerns was not associated (effect-size 0.12, p = 0.508, data not shown). Of the eight items related to clinical training, concerns about infecting others during training was positively and significantly associated with SCL-5 (effect-size for neither disagree nor agree versus strongly agree = 0.26, *p* = 0.005, data not shown).

**Table 3:** Hierarchical regression analysis of factors associated with psychological distress and overall quality of life in all students (*N*=2605)

		Psychological distress (SCL-5)^**a**^			Overall quality of life		
Variable	n (%)	Adjusted coefficient	95% Confidence Interval	*P*-value	Adjusted coefficient	95% Confidence Interval	*P*-value^b^
**Concerns about the quality of education**							
Strongly agree	1196 (46)	Ref.		**0.031**	Ref.		0.722
Agree	869 (33)	-0.06	-0.13, 0.01		0.01	-0.06, 0.09	
Neither disagree nor agree	287 (11)	-0.13	-0.24, -0.03		-0.01	-0.12, 0.10	
Disagree	160 (6)	-0.15	-0.28, -0.02		0.05	-0.09, 0.19	
Strongly disagree	94 (4)	-0.17	-0.33, 0.00		0.11	-0.06, 0.29	
**Satisfaction with the curriculum**							
Strongly agree	274 (11)	Ref.		**<0.001**	Ref.		**<0.001**
Agree	1108 (43)	0.06	-0.05, 0.17		-0.18	-0.30, -0.06	
Neither disagree nor agree	731 (28)	0.16	0.03, 0.18		-0.37	-0.50, -0.24	
Disagree	365 (14)	0.25	0.11, 0.40		-0.56	-0.71, -0.41	
Strongly disagree	128 (5)	0.39	0.20, 0.58		-0.87	-1.07, -0.67	
**Trust in governmental handling of the COVID-19 situation**							
Strongly disagree	154 (6)	Ref.		**<0.001**	Ref.		**< 0.001**
Disagree	77 (3)	-0.03	-0.24, 0.18		-0.11	-0.34, 0.11	
Neither disagree nor agree	562 (22)	-0.16	-0.30, -0.02		0.06	-0.09, 0.20	
Agree	1344 (52)	-0.24	-0.37, -0.11		0.17	0.04, 0.31	
Strongly agree	469 (18)	-0.30	-0.44, -0.16		0.23	0.08, 0.39	
**Trust in universities’ handling of the COVID-19 situation**							
Strongly disagree	181 (7)	Ref.		0.271	Ref.		**0.001**
Disagree	447 (17)	0.06	-0.09, 0.20		-0.18	-0.33, -0.02	
Neither disagree nor agree	783 (30)	0.08	-0.06, 0.23		-0.26	-0.41, -0.11	
Agree	982 (38)	0.01	-0.13, 0.16		-0.13	-0.29, 0.02	
Strongly agree	213 (8)	0.10	-0.08, 0.28		-0.27	-0.46, -0.08	
**Age category**							
<25	1845 (71)	Ref.		**<0.001**	Ref.		**0.030**
25-29	377 (15)	-0.05	-0-14, 0.03		0.05	-0.04, 0.15	
30+	382 (15)	-0.23	-0.32, -0.15		0.16	0.07, 0.25	
**Feeling lonely due to COVID-19**							
Strongly disagree	164 (6)	Ref.		**<0.001**	Ref.		**<0.001**
Disagree	380 (15)	0.16	0.02, 031		-0.30	-0.45, -0.15	
Neither disagree nor agree	444 (17)	0.27	0.13, 041		-0.45	-0.60, -0.30	
Agree	899 (35)	0.52	0.39, 0.65		-0.80	-0.94, -0.66	
Strongly agree	718 (28)	0.95	0.82, 1.09		-1.35	-1.49, -1.20	
**Fear of COVID-19 (continuous z-score)**	2605 (100)	0.47	0.43, 0.51	**<0.001**	-0.09	-0.13, -0.06	**<0.001**
**Risk of COVID-19**							
No	2089 (80)				Ref.		**0.022**
Uncertain	327 (13)				-0.12	-0.22, -0.02	
Yes	189 (7)				-0.10	-0.23, 0.02	

^a^The Hopkins Symptom Checklist; ^b^*P*-values below 0.05 in bold

### Impact of satisfaction and quality concerns on quality of life

Similarly, the association of student reported satisfaction and concerns about educational quality on QOL were explored in all students and those engaged in clinical training in two separate hierarchical regression models. For all students, satisfaction with the curriculum was positively and significantly associated with QOL (effect-size for strongly disagree versus strongly agree = 0.87, *p*<0.001, Table 3). For students involved in clinical training during the pandemic, satisfaction retained a significant association (effect-size 0.85, *p*<0.001), whereas neither educational concerns nor any of the items related to clinical training were associated with QOL (data not shown).

## Discussion

At the peak of the second wave of the COVID-19 pandemic, we investigated Norwegian baccalaureate nursing students’ satisfaction with their educational curriculum and their concerns about the quality of their education, with emphasis on the conduct of clinical training. Compared to pre-pandemic reference data from the same institutions, satisfaction was lower, but with a small effect-size. Most students reported to be concerned about the quality of their education, influenced also by worries about conduct of clinical training. Satisfaction and concerns about quality were strongly associated with trust in universities’ handling of the pandemic. Educational satisfaction positively affected overall QOL, and to a lesser degree, psychological health, whereas concerns about quality only affected psychological health.

We aimed to determine educational factors that could explain reduced overall QOL and increased psychological distress, previously demonstrated in nursing students during the second wave of COVID-19 in Norway [3]. This analysis was prompted by findings that fear of COVID-19 was strongly associated with psychological distress, but only moderately accounted for the decrease in overall QOL in these students. Assessed by one claim, *I am, overall, satisfied with the curriculum I am currently attending*, we chose to evaluate satisfaction as it has been used in an identical manner in Norwegian students for many years prior to the pandemic, and representative data was available for comparison [17]. A major finding of our study was the relatively small decline in student satisfaction from pre-covid reference levels. Others have also analyzed nursing students’ level of satisfaction during the pandemic, mostly satisfaction with remote learning [33, 34] or imposed changes in clinical placement [34], but without comparisons to pre-pandemic levels of overall satisfaction.

We found trust in universities’ handling of the pandemic to be the major determinant of satisfaction, complying with studies suggesting that universities’ instructional effectiveness and student support facilities may influence overall satisfaction under normal circumstances [11, 14, 15]. Our data suggest that building trust may be important and that measures taken during a crisis need to be clear and understandable to sustain satisfaction. When analyzing satisfaction of nursing students with remote learning situations specifically, also institutions/’instructors’ attitudes towards these teaching situations and optimal use of technology were main determinants of satisfaction during the pandemic [33]. Thus, institutional handling of the pandemic, as any future crisis affecting nursing education, seems to play a major role in maintaining satisfaction.

Students’ satisfaction with the curriculum has attracted interest in research of higher education even before the COVID-19 pandemic [11, 35]. Satisfaction of undergraduate nursing students is important both for attracting students to a health profession, for their motivation to complete the education and enter a professional career as much needed nurses [36]. Of interest, the perceived quality of the education, and by inversion, concerns about a lack thereof, have been invoked as one of several factors influencing satisfaction [16]. Unfortunately, to our knowledge, no data with a similar question as ours, *Due to the COVID-19 pandemic I am concerned that the quality of my education will be poorer than it would otherwise have been*, is available to directly assess the level of change in worries that nursing students have experienced because of the pandemic. We found however, that in addition to a major impact of trust in universities’ handling of the COVID-19 crisis, concerns about overall educational quality were associated with satisfaction. With 61% of students in our sample having participated in at least some clinical training during the pandemic, their concern about the conduct of this activity affected the overall educational satisfaction only to a small extent (increase in explained variance of the models, Nagelkerke pseudo R^2^, from 0.41 to 0.44). This may have to do with policies adopted by Norwegian universities, where clinical training in nursing homes and hospitals continued with precautionary regulations even before large-scale vaccination of the elderly population and health care professionals occurred. This reassuring finding of ours contrasts others where concerns about the clinical training have impacted overall perception of their education [37]. This may not come as a surprise since it may be particularly difficult to replace face-to-face “hands-on” training during a pandemic [34, 38]. Furthermore, higher levels of concern reported in first year students suggest better strategies may be needed to take care of certain groups of students. A Danish study of health profession students also reported young age, female sex and enrollment in baccalaureate curriculums to be associated with higher levels of educational stress during the pandemic, all characteristics typical for undergraduate nursing students [10].

In our sample, satisfaction with the educational curriculum was associated with overall QOL with a large effect-size in regression models, to an extent comparable with the effect of loneliness [3]. The effect of satisfaction on psychological distress was lower in magnitude, but these findings together indicate that universities may contribute to nursing students overall QOL, and thereby possibly to their academic performance. Efforts to build effective curriculums of high academic quality seem to be important, especially during a crisis. Overall concerns and those specifically related to disruptions of clinical training, were associated only with psychological distress. Thus, nursing students’ concerns about the educational quality seem to target specifically the conduct of education and does not affect the more general level of QOL in our sample.

The COVID-19 pandemic with different variants of the virus has hit the world in several waves over the last two years. Nevertheless, at some point students’ lives will return to a new state of normal where educational curriculums begin to transit back to the modalities previously used. Thus, time is due for institutions of higher education to consider structures and strategies that support students’ psychological health and educational trajectory during current and future pandemics or similar crises. In this regard, findings from the present study suggest that maintaining and building trust are important to improve student satisfaction, reduce educational quality concerns and benefit students’ overall QOL. Although not directly assessed in our work, pre-existing procedures for risk-adapted educational instruments (online learning, simulation training on campus, infection control measures during clinical training etc), as well as student counseling and transparent communication may well build trust in the baccalaureate nursing education.

### Strengths and limitations

The present survey is based on cross-sectional data and does not allow assessments of change over time at an individual level. However, available reference data derived from two National Student Surveys (prior to and between the first and second wave of the COVID-19 pandemic) [17] add strength to our study by providing measures of student satisfaction at three different time-points for comparison. Although varying across the five participating institutions, the overall response rate of 43% compares favorably to what is commonly achieved in electronic surveys [39], and reaches the same level as the Norwegian student surveys used here for comparison [40, 41].

Furthermore, the large sample of baccalaureate nursing students from universities using a curriculum comparable to most European and many international recommendations [20], increases the external validity of our findings. The narrow focus on clinical training, not covering other COVID-19 related educational changes, for example students’ perceptions of transition to digital learning, represents a limitation of our study.

## Conclusion

Baccalaureate nursing students’ satisfaction and concerns about educational quality were strongly associated with trust in universities’ handling of the pandemic. Overall level of concern about the quality was moderately affected by concerns related to the conduct of clinical training, whereas student satisfaction was not. Furthermore, students’ satisfaction may significantly impact perceived overall QOL, and to a lesser degree, psychosocial health, whereas concerns about quality only affected psychological health.

## Data Availability

Access to the dataset supporting the results reported will be made available by corresponding author on editor or reviewers’ request.
